# Stability and safety of transbronchial dye mixture for preoperative localization in a porcine model

**DOI:** 10.1111/1759-7714.14814

**Published:** 2023-02-01

**Authors:** Wan Ho Yoo, Sae Rom Kim, Soo Han Kim, Jongggeun Lee, Jeongha Mok, Dong Hoon Shin, Hyo Yeong Ahn, Jung Seop Eom

**Affiliations:** ^1^ Department of Internal Medicine Pusan National University Hospital Busan South Korea; ^2^ Department of Thoracic and Cardiovascular Surgery Pusan National University Hospital Busan South Korea; ^3^ Department of Pathology Pusan National University Yangsan Hospital Yangsan South Korea; ^4^ Biomedical Research Institute Pusan National University Hospital Busan South Korea

**Keywords:** dye mixture, indigo carmine, lung nodule, stability, transbronchial injection

## Abstract

**Objective:**

For thoracoscopy, the usefulness of a dye mixture of indigo carmine and Lipiodol for localizing lung lesions has been reported. However, little is known about the stability and safety of this dye mixture injected on the visceral pleura through a bronchoscope.

**Methods:**

Porcine models were divided into three groups according to the detection time of the dye mixture: group A with a detection time of 4 h; group B, 8 h; and group C, 24 h. A dye mixture of indigo carmine and Lipiodol (0.5 mL each) was sprayed onto the visceral pleura both in the ventral and dorsal regions via a spray catheter.

**Results:**

Twelve markings were created on the visceral pleura of the porcine lung (six ventral and six dorsal) in the six porcine models. At predetermined detection times, all 12 dye markings (100%) were visible on the visceral pleura. The mean longest diameter of the dye marking in the ventral and dorsal regions was 18.8 mm and 24.3 mm, respectively. In groups B and C, pathological changes in the lymphatic system, such as lymphatic dilatations, were found; minimal changes were found in group B, however, these changes with oval‐shaped lymphatic cysts and Lipiodol accumulation, were more evident in group C.

**Conclusions:**

The dye mixture of indigo carmine and Lipiodol had reliable stability and visibility. In terms of safety, it may be necessary to check the dye mixture on the lung surface within 8 h.

## Graphical abstract

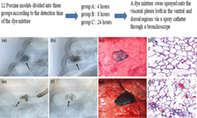

## INTRODUCTION

As low‐dose computed tomography (CT) has become a standard screening method for lung cancer in high‐risk populations, the detection of early‐stage lung cancer has increased.^1^ However, it is challenging for pulmonary physicians to determine whether the pulmonary nodule found on a CT scan is malignant, leading to a surge in demand for surgical biopsy of pulmonary nodules and sequential lobectomy.^2^ Some pulmonary nodules, located in the peripheral lung parenchyma and attached to the visceral pleura, can be easily detected by a thoracic surgeon during thoracoscopic surgery without preoperative preparation. However, 14.1% of lung nodules are more centrally located, requiring preoperative or intraoperative procedures to locate the target pulmonary nodules.^3^

Various methods for the localization of pulmonary nodules have been introduced for thoracoscopic surgery, such as CT‐guided insertion of localizer, percutaneous injection of dye material, and electromagnetic navigation bronchoscopy. However, there is no standard technique yet.^4^, ^5^, ^6^, ^7^, ^8^ CT‐guided percutaneous localization is a well‐known method and shows a high success rate. However, there are concerns of mechanical complication, such as pneumothorax and air embolism, which can be fatal.^5^, ^7^ In contrast, transbronchial dye marking using flexible bronchoscopy is known as an accurate and safe procedure for locating the target pulmonary lesions.^9^ Recently, we have reported the usefulness of a dye mixture of indigo carmine and Lipiodol.^10^, ^11^ However, little is known about its stability and safety when injected on the visceral pleura through a bronchoscope. We performed an animal experiment to evaluate the stability and safety of indigo carmine and Lipiodol dye mixture injected on the visceral pleura of a porcine model, using bronchoscopy.

## METHODS

### Animal models and study groups

This study was approved by the Institutional Animal Care and Use Committee of Pusan National University Hospital (no. PNUH‐2022‐199). Six live female pigs were divided into three groups according to the detection time of the dye mixture: group A, 4 h; group B, 8 h; and group C, 24 h.

### Animal experiment

General anesthesia was induced using intravenous alfaxalone and vecuronium and intramuscular xylazine. After anesthesia induction, the pigs were placed on a surgical bed, and endotracheal intubation was performed. While connected to a mechanical ventilator, general anesthesia was maintained using inhaled halothane. Tracheostomy was performed using the percutaneous dilatational technique,^12^ and an endotracheal tube was inserted into the distal trachea through the tracheostomy site.

Before bronchoscopy, indigo carmine and Lipiodol (0.5 mL each) were mixed using two syringes and a 3‐way stopcock. A flexible bronchoscope (BF‐260; Olympus) was introduced into the ventral and dorsal areas of the lung through the endotracheal tube, and a spray catheter (PW‐6P‐1; Olympus) was inserted into the periphery of the lung through the working channel of the bronchoscope under fluoroscopic guidance. After confirming that the distal end of the spray catheter was positioned around the visceral pleura in the ventral region of the lung, 1 mL dye mixture with 2 mL air was sprayed into the visceral pleura by the spray catheter. The same procedure was repeated in the dorsal region. If the dye mixture was not clearly visible on fluoroscopy, an additional 2–4 mL air was sprayed by the spray catheter at the investigator's discretion.

At the predetermined detection time‐point for each study group, fluoroscopic images were checked again, and thoracotomy was performed. Before thoracotomy, the endotracheal tube was inserted into the main bronchus contralateral to the target lung to maintain one‐lung ventilation. After thoracotomy, the visibility of the dye mixture on the visceral pleura was evaluated, and its size was calculated. Finally, lung tissues were excised and collected for histological examination.

### Study outcomes

The primary outcome was the visibility of the dye mixture on the visceral pleura identified after thoracotomy. The secondary outcomes were as follows: (1) fluoroscopic visibility, (2) extent of dye marking on the visceral pleura, calculated by multiplying the longest diameter by the vertical diameter, and (3) histologic findings. Histology of the excised lung tissue was scored 0, 1, and 2 using the lung injury scoring system with five parameters^13^ as follows: (1) neutrophils in the alveolar space, (2) neutrophils in the interstitial space, (3) presence of a hyaline membrane, (4) proteinaceous debris filling the airspaces, and (5) alveolar septal thickening (Appendix A, Table A1). The sum of the scores for these five variables was calculated using the formula of the lung injury scoring system.

### Statistical analysis

Continuous variables were expressed as medians with ranges and compared using the Kruskal–Wallis test. Statistical significance was set at *p* < 0.05. All analyses were performed using SPSS Version 24.0 for Windows (SPSS).

## RESULTS

A total of 12 markings were created on the visceral pleura of the lungs (six ventral and six dorsal) using a dye mixture of indigo carmine and Lipiodol in the six porcine models. The median age and weight of the pigs were 13 weeks (range, 12–14) and 36.5 kg (range, 35–41), respectively. The median bronchoscopy and thoracotomy times were 8.6 min (range, 6.5–10.5) and 29.8 min (range, 26–35.5), respectively.

### Visibility and extent of dye‐stained area

**FIGURE 1:**
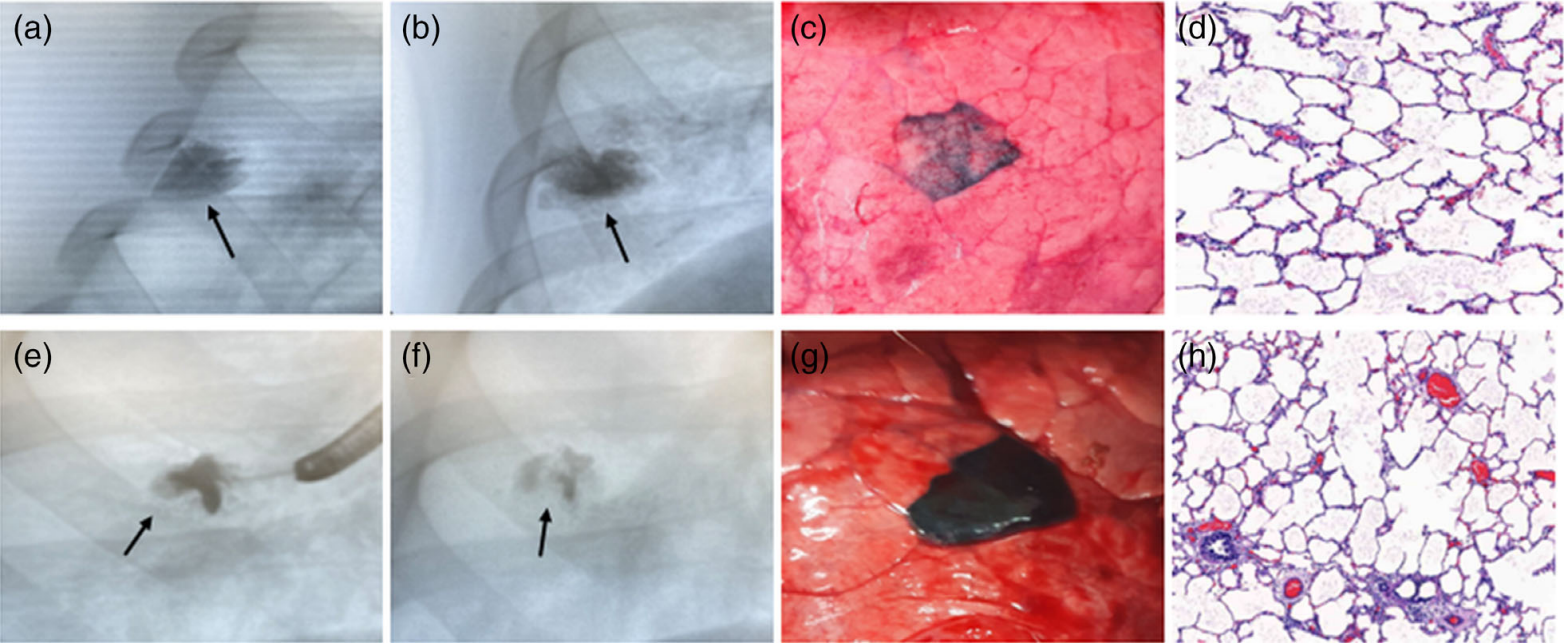
Representative cases of fluoroscopy for bronchoscopic dye mixture detection in the porcine lung. Photographs (a), (b), (c), and (d) are of group A and (e), (f), (g), and (h) of group B. Fluoroscopy performed after dye mixture (arrow) injection (a) and (e). Fluoroscopy performed just before thoracotomy (b) and (f). A dye mixture on the visceral pleura of a porcine model identified after thoracotomy (c) and (g). Histopathologic findings of the marking regions of the porcine lung (d) and (h)

At the predetermined detection time‐points in three study groups, all 12 dye markings (100%) were visible on the visceral pleura (primary outcome) (shown in Figure 1). The mean longest diameters of the dye marking in the ventral and dorsal regions were 18.8 mm (range, 13–30) and 24.3 mm (range, 15–35 mm), respectively (Table 1). No differences in the longest diameter of the dye marking in the ventral (*p* = 0.37) and dorsal regions (*p* = 0.71) was observed between the three study groups. The mean extent of the dye marking in the ventral and dorsal regions was 247 mm^2^ (range, 130–300) and 406 mm^2^ (range, 198–660), respectively. No difference in the extent of the dye marking in the ventral (*p* = 0.23) and dorsal regions (*p* = 0.57) was observed between the three study groups.

**TABLE 1:** Longest diameters and extents of dyed area according to the detection time

Variables	Group A		Group B		Group C		*p*‐value
	Pig 1	Pig 2	Pig 3	Pig 4	Pig 5	Pig 6	
Longest diameter							
Ventral region, mm	13	17	18	20	15	30	0.37
Dorsal region, mm	35	18	18	30	30	15	0.71
Extent of dye marking^a^							
Ventral region, mm^2^	130	255	270	300	225	300	0.23
Dorsal region, mm^2^	595	216	198	540	660	225	0.57

*Note*: The detection times for groups A, B, and C were 4 h, 8 h, and 24 h, respectively.

^a^ The extent was calculated by multiplying the longest diameter by the vertical diameter.

### Fluoroscopic visibility

All 12 dye markings were detectable on fluoroscopy immediately after their injection and at each detection time‐point (shown in Figure 1).

### Histopathologic findings

**FIGURE 2:**
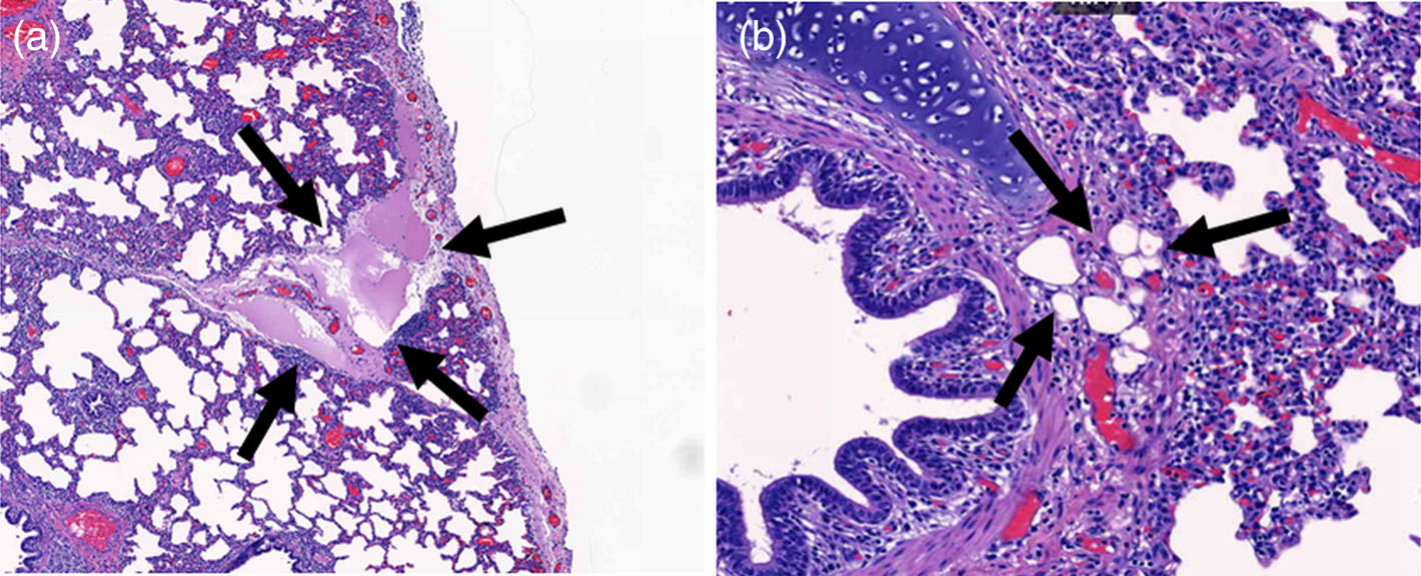
Photomicrograph findings of lung specimen in group C. Lymphatic dilatation (a), Lipiodol accumulation in cystic change (b)

According to the formula of the lung injury scoring system, no parenchymal lung injury, such as neutrophil infiltration, hyaline membrane, proteinaceous debris filling the airspaces, or alveolar septal thickening, were noted in all specimens of the three study groups (shown in Figure 1). No histological changes were observed in the lymphatics in group A; however, lymphatic dilatation was found in groups B and C (shown in Figure 2(a)). The extent of lymphatic dilatation was <1% of each specimen slice in group B and <5% in group C. Particularly, oval‐shaped lymphatic cysts with Lipiodol accumulation were found in group C (shown in Figure 2(b)).

## DISCUSSION

As there is a clear difference in the overall survival according to TNM stage, early detection and complete resection are considered the most important strategies for lung cancer treatment.^14^ In particular, with the recent widespread use of low‐dose CT scans for lung cancer screening, the detection of small lung nodules with suspected lung cancer has been increasing. Therefore, precise localization of lung nodules has become more important for thoracoscopic wedge resection and sequential lobectomy.^1^, ^2^ We have previously reported that transbronchial injection of a dye mixture of indigo carmine and Lipiodol can be a good method for preoperative marking of the visceral pleura.^11^, ^15^ In the present study, using a porcine model, we demonstrated that this dye mixture, injected using a bronchoscope and spray catheter, remained on the visceral pleura for up to 24 h and could be detected with the naked eye. To the best of our knowledge, this is the first study to evaluate the stability of a dye for preoperative marking of the visceral pleura.

Unlike long‐lasting metal coil implantation, dye injection for preoperative marking is associated with the following limitations. When a water‐soluble dye material, such as methylene blue, indigo carmine, or indocyanine blue, is injected into the lung, it is expected to spread on the lung surface in a short time.^9^, ^16^, ^17^ Previous studies have shown that dyes injected into the visceral pleura are easily diffused and quickly removed.^18^, ^19^ Moreover, in anthracosis, pleural adhesions, and emphysematous lungs, a dye disappears more rapidly from the lungs.^9^, ^16^ To overcome these limitations of dye diffusion and disappearance, fat‐soluble Lipiodol was added to indigo carmine and mixed to prepare fine particles using two syringes and a stopcock in this study. As a result, all dye markings were visible on the visceral pleura in both ventral and dorsal regions of the porcine lungs for up to 24 h. Our results suggest that mixed fine particles of indigo carmine and Lipiodol diffuse and are removed more slowly than the water‐soluble dye alone.

Although transbronchial dye injection is a simple and easy technique to locate peripheral lung lesions, there have been concerns regarding detection failure. Despite that Lipiodol itself cannot be detected by direct vision, Lipiodol not only helps prevent the spread of dye mixtures, but can also be used to locate peripheral lung lesions through fluoroscopy if dye detection fails on the visceral pleura. In the present study, all dye markings were observed under fluoroscopy at the detection time‐point in all three study groups, suggesting that fluoroscopic searching can be an alternative method in case of detection failure.

Several studies have suggested that dye may lead to destruction or histopathological changes in the lung tissue. Materials, such as barium, show harmful effects through lung inflammation on pathologic analysis.^20^, ^21^ In our study, no harmful histological changes were observed in the lung parenchyma. However, because the detection time was prolonged, lymphatic dilatations were observed with minimal changes in group B and more evident in group C. In addition, oval‐shaped lymphatic cysts, presumably Lipiodol accumulation, were observed in the specimens of group C. These findings implied that the Lipiodol accumulation blocked the lymphatic system in the porcine lungs, leading to lymphatic dilatation and cyst formation. In this regard, a duration of >8 h after dye injection may induce lymphatic damage and worsen over time. In terms of both safety and stability, reliable results were obtained when the lung specimens were checked within 8 h.

Mechanical complications, such as pneumothorax, parenchymal hemorrhage, and air embolism, are well‐known adverse effects of CT‐guided percutaneous approach.^8^, ^17^, ^21^ Previous animal studies reported acute lung injury after CT‐guided percutaneous localization proven by histopathologic examination.^21^ However, bronchoscopic approach has appeared to be associated fewer complications and competitive yield.^22^ Given safety issues of percutaneous approach, we performed bronchoscopic approach with dye mixture and no serious complications were observed.

This study had several limitations. First, it included a small number of animals. Second, we used a 1:1 mixture of indigo carmine and Lipiodol; however, there was no evidence of the relevance of the dye mixture ratio. Further clinical studies are required to evaluate the optimal proportions of dye mixtures. Third, this study was an animal experiment, and it is difficult to apply our results directly to humans.

## CONCLUSION

In conclusion, the dye mixture of indigo carmine and Lipiodol had reliable visibility. The dye mixture lasted for 24 h and could be identified by both the naked eye and fluoroscopy. However, in terms of safety, it may be necessary to check the dye mixture on the lung surface within 8 h.

## AUTHOR CONTRIBUTIONS

*Study conception and design*: Wan Ho Yoo, Hyo Yeong Ahn, and Jung Seop Eom. *Data analysis*: Sae Rom Kim, Soo Han Kim, and Dong Hoon Shin. *Data interpretation and manuscript writing*: Jongggeun Lee and Wan Ho Yoo. *Revision of manuscript and contribution of intellectual content*: All authors. All authors have read and agreed to the published version of the manuscript.

## CONFLICT OF INTEREST STATEMENT

The authors have no conflicts of interest to declare.

## Data Availability

All data generated or analyzed during this study are included in this published article.

## APPENDIX A

**TABLE A1:** Lung injury scoring system

	Score per field		
	0	1	2
A. Neutrophils in the alveolar space	None	1–5	>5
B. Neutrophils in the interstitial space	None	1–5	>5
C. Hyaline membranes	None	1	>1
D. Proteinaceous debris filling the airspaces	None	1	>1
E. Alveolar septal thickening^a^	<2×	2 × −4×	>4×

*Note*: Sum of scores = [(20 × A) + (14 × B) + (7 × C) + (7 × D) + (2 × E)]/(no. of fields × 100).

^a^ Septal thickness was evaluated as septal thickening when it was twice as large as normal.
