# Correction to “Stability and safety of transbronchial dye mixture for preoperative localization in a porcine model”

**DOI:** 10.1111/1759-7714.14934

**Published:** 2023-05-22

Yoo WH, Kim SR, Kim SH, Lee J, Mok J, Shin DH, et al. Stability and safety of transbronchial dye mixture for preoperative localization in a porcine model. Thorac Cancer. 2023;14(9):834–9. https://doi.org/10.1111/1759-7714.14814

The authors would like to update affiliation 2. The correct affiliation is shown below:

^2^Department of Thoracic and Cardiovascular Surgery, Pusan National University School of Medicine, Pusan National University Hospital, Busan, South Korea.

We apologize for these errors.

